# Prognostic value of magnetic resonance imaging combined with electromyography in the surgical management of cervical spondylotic myelopathy

**DOI:** 10.3892/etm.2013.934

**Published:** 2013-01-30

**Authors:** FA-JING LIU, YA-PENG SUN, YONG SHEN, WEN-YUAN DING, LIN-FENG WANG

**Affiliations:** Department of Spine Surgery, The Third Hospital of Hebei Medical University, Shijiazhuang 050051, P.R. China

**Keywords:** cervical spondylotic myelopathy, electromyography, magnetic resonance imaging, prognosis

## Abstract

The present study aimed to evaluate the value of pre-operative magnetic resonance imaging (MRI) combined with electromyography (EMG) for predicting clinical outcome following surgical management of cervical spondylotic myelopathy (CSM). A total of 94 patients with cervical compressive myelopathy were prospectively enrolled and treated with anterior, posterior and posterior-anterior united decompression between October 2007 and February 2009. Prior to surgery 1.5-T MRI and EMG were performed in all patients. The patients were classified into four types based on the presence (+) or absence (−) of an increased signal intensity (ISI) on the T2-weighted magnetic resonance (MR) images and also based on the positive (+)/negative (−) results of the EMG. The four types were as follows: Type I, MRI/EMG (−/−); Type II, MRI/EMG (+/−); Type III, MRI/EMG (−/+); and Type IV, MRI/EMG (+/+). The clinical outcome was also graded according to a modified Japanese Orthopedic Association (JOA) scoring system. Furthermore, pre- and post-operative clinical data were statistically analyzed to explore the correlation between the factors. There were 36 cases (38%) of Type I, 16 (17%) of Type II, 13 (14%) of Type III and 29 (31%) of Type IV. According to the analysis of the clinical data between the four types, there were significant differences in the disability classifications, pre-operative JOA scores and disease duration (P<0.05), but there were no significant differences in gender, age or cord compression ratios (P>0.05). Until the final follow-up, there was a significant difference in the recovery ratio between the four study groups (Hc=27.46, P<0.05). Further comparison showed that the surgical outcome was best in Type I patients and worst in Type IV patients. In conclusion, there was a distinct correlation between classification and the rate of clinical improvement. Patients who had a negative EMG and those without an ISI on T2-weight images tended to suffer only mild symptoms, a short disease duration and, most significantly, experience a good surgical outcome.

## Introduction

Cervical spondylotic myelopathy (CSM) is a chronic and devastating disease that may be improved by surgical decompression in certain patients. In other patients, surgical intervention does not change their neurological condition or the natural history of the disease. Factors that affect the prognosis and the results of decompressive surgery have been investigated by numerous authors. The increased signal intensity (ISI) of the spinal cord in magentic resonance imaging (MRI) of CSM is thought to reflect the pathological changes in the spinal cord and be indicative of the prognosis (1). However, the significance of intramedullary signal changes in predicting the surgical outcome is greatly controversial (2,3). As it is impossible to quantify the extent of the dysfunction of the spinal cord from physiological findings, electrophysiological findings have been used (4). The purpose of the present study was to elucidate whether changes in the the magnetic resonance (MR) T2 image signal combined with the electromyography (EMG) index reflect the severity of the symptoms and form a prognosis for patients with CSM.

## Patients and methods

### Patients

Between October 2007 and February 2009, 94 patients with CSM whose diagnoses were confirmed by clinical and image examination were examined by EMG in the Third Hospital of Hebei and were enrolled in the present study. These examinations were performed to exclude peripheral nerve injuries, including cubital tunnel syndrome and carpal tunnel syndrome. Patients with traumatic cervical cord injuries without bony lesions were also excluded. The group consisted of 56 males and 38 females with a mean age of 52.3 years (range, 46–69 years). The mean disease duration prior to surgery was recorded as 10.5 months (range, 3–32 months). All patients had been evaluated clinically. The patients were diagnosed on the basis of the symptoms and signs of CSM, including loss of sensitivity in the hands and/or feet. Several patients had increased reflexes in the knee and ankle. Certain patients presented with stance ataxia and gait unsteadiness in addition to a spastic gait. The patients were treated with anterior, posterior or posterior-anterior united decompression according to their condition. A total of 67 patients were operated on using anterior surgery, 15 patients using posterior surgery and 12 patients using the posterior-anterior combined approach. The study was approved by the HeBei Medical University, Shijiazhuang, China. Written informed patient consent was obtained from the patient.

### MRI evaluation

All patients underwent high-resolution MRI with a 1.5-T (Siemens Magnetom Symphony, Malvern, PA, USA) imager prior to surgery. T1- and T2-weighted images of sagittal views of the cervical cord were obtained using a spin echo sequence system for the T1-weighted images and a fast spin echo sequence system for the T2-weighted images. A surface coil was also used. The slice width was 4 mm and the acquisition matrix was 512×256. The sequence parameters included a repetition time (TR) of 612 msec/echo time (TE) of 13 msec for the T1-weighted images and a TR of 2400 msec/TE of 114 msec for the T2-weighted images. Based on the MRI, the patients were divided into 2 groups, consisting of those patients with (+) and without (−) an ISI on the T2-weighted MR images. These classifications were decided upon by two independent radiologists who were experienced in spinal imaging. The concordance rate between the two observers in evaluating the signal change on the T2-weighted images was 92.1% and κ-coefficient was 0.89. Values of the κ-coefficient close to 1.0 indicated good reproducibility. The two observers established the final classification by consensus.

### EMG evaluation

EMG was performed with a Nicolet Viking IV EMG Unit (Nicolet Biomedical, Madison, WI, USA) prior to surgery. The first dorsal interosseous, extensor digitorum communis, pronator teres, biceps brachii, deltoid, sternocleidomastoideus, brachioradialis and the short abductor thumb muscles were sampled in an assessment of spontaneous activity, motor unit action potential (MUAP) parameters and interference patterns. The motor and sensory nerve conduction velocities of the median, ulnar, radial and axillary nerves were also detected. Using EMG, electrical potential changes of the muscles and the motor and sensory nerve conduction velocities of the peripheral nerves were investigated with aggregate analyses of relaxation, slight constriction and gross constriction, respectively. Presence of a lengthened duration (exceeding the normal value by 20% in the same age group), large-amplitude MUAPs and polyphasic motor units in at least two myotomes unilaterally or bilaterally were regarded as a positive result (+) due to an anterior horn cell lesion resulting from CSM (5,6). Otherwise, the EMG results were normal (−).

### Clinical assessment

Patients were classified into four types according to the results of their MRI and EMG: Type I, MRI/EMG (−/−); Type II, MRI/EMG (−/+); Type III, MRI/EMG (+/−); and Type IV, MRI/EMG (+/+). The disability classification of CSM depended on a grading system that emphasized gait abnormalities (Table I) (7). The severity of pre-operative and post-operative neurological deficits was scored according to a modified Japanese Orthopedic Association (JOA) scoring system (highest score, 17 points) (8). To rate the surgical outcome, a recovery rate was calculated using the following formula: Recovery rate (%) = (post-operative JOA score − pre-operative JOA score)/(17 − pre-operative JOA score) × 100. The surgical outcome was further quantified by categorizing the recovery rate percentage into 4 groups (9): excellent, from 75 to 100%; good, from 50 to 74%; fair, from 25 to 49%; and poor, from 0 to 24%.

### Clinical data

Deformity of the spinal cord was expressed by the ratio of the sagittal diameter divided by the transverse diameter of the spinal cord observed on the T1-weighted transverse images (cord compression ratio = sagittal diameter/transverse diameter). The cord compression ratio was evaluated according to the method previously presented by Nagata *et al* (<1/3, 1/3–1/2 and >1/2) (10).

The radiological findings of all patients were evaluated and recorded in detail. The clinical parameters, including age, gender and duration of symptoms, were also recorded.

### Statistical analysis

SPSS 13.0 software (Chicago, IL, USA) was used for statistical analysis. Values are presented as the mean ± standard deviation (SD). A non-parametric analysis using the Kruskal-Wallis H test was used to analyze the differences among the four classification types, while the Wilcoxon test was used to analyze the differences between two types. The association between the four types and the other variables was assessed by χ^2^ and F tests. P<0.05 was considered to indicate a statistically significant difference.

## Results

The mean follow-up period among the patients was 23 months (range, 12–36 months) subsequent to surgery. No patients required additional cervical decompressive surgery due to restenosis or residual stenosis. Pre-operative MRI showed an alteration of the intramedullary signal on the T2-weighted MR images in 45 of 94 cases (48%). EMG was abnormal in 42 of 94 cases (45%). The results revealed that 36 cases (38%) were classified as Type I, 16 (17%) as Type II, 13 (14%) as Type III and 29 (31%) as Type IV.

For the pre-operative CSM disability grading, 17 cases (18%) were categorized as grade 0, 19 as grade 1 (20%), 17 as grade 2 (18%), 16 as grade 3 (17%), 14 as grade 4 (15%) and 11 as grade 5 (12%). The Kruskal-Wallis H test showed a significant difference in the disability grading between the four study groups (Hc=9.000, P<0.05; Table II). The surgical outcome was classified as excellent in 41 patients, good in 22 patients, fair in 20 patients and poor in 11 patients. The Kruskal-Wallis H test showed a significant difference in the recovery ratio between the classifications (Hc=27.46, P<0.05). The Wilcoxon test showed that the surgical outcome was best in patients of Type I and worst in those of Type IV. There was no significant difference in the recovery ratio between Types II and III (Table III).

The analysis of variance for the factors of age (P=0.131), disease duration (P= 0.004) and pre-operative JOA score (P= 0.017) among the four types showed significant differences. The Student-Newman-Keuls (SNK) test analyzed the disease duration and showed significant differences between type I and II or III (P<0.05), type IV and II or III (P<0.05) and type I and IV (P<0.05). However, types II and III showed no significant difference (P>0.05). The SNK test analyzed the pre-operative JOA score and showed the same statistical results (Table IV).

The Pearson χ^2^ test performed a gender anaysis between the four types and showed no significant differences (P=0.910). The Kruskal-Wallis H test analyzed the cord compression ratio and also showed no significant differences between the four types (P=0.671; Table V).

## Discussion

An ISI on the T2-weighted MR images is often observed in patients with CSM and therefore the relevance of a pre-operative ISI on T2-weighted MR imaging has been extensively investigated. A number of authors have performed a series of studies on the association between ISI and surgical outcomes. Certain authors reported that patients with ISI have poor prognosis following surgery (11,12), particularly those with high signal intensity ratios in the T2-weighted MR images (13,14). Other studies have suggested that an ISI did not predict a poor surgical outcome in CSM (3,15). In studies of histopathological examination and MRI, ISI without signal changes on the T1-weighted images appeared non-specifically in patients with mild lesion alterations, including the loss of nerve cells, gliosis, edema in the gray matter, Wallerian degeneration, demyelination and edema in the white matter (1). al-Mefty *et al* reported that intramedullary changes of the spinal cord observed using MRI coincided with pathological changes in the gray matter of the spinal cord, which included the loss of motor neurons, necrosis and cavitation in experimental chronic compressive myelopathies of canines (16). Therefore, the present study suggested that the signal intensity changes in MRI were able to reflect the condition of the spinal cord, but that use of only an ISI on the T2-weighted images may not exactly reflect the real neural situations of the CSM patients, and it would be more objective to combine it with the EMG examination.

Due to the compression of intra-spinal protuberances, CSM may lead to devastating and crippling neurological deficits, which are mostly detected by EMG through the abnormal stimulation of the nerve fibres travelling along the motor or sensory fascicles (10). EMG is useful for evaluating any root involvement and may be required for the exclusion of differentials, including amyotrophic lateral sclerosis and even subacute combined degeneration of the spinal cord (4,17) prior to the diagnosis of CSM. In quantitative analysis, MUAP duration is the most robust feature used to differentiate between myogenic and neurogenic conditions (18). It is usual to encounter enlarged MUAPs in patients with neurogenic diseases (5), including pathological changes in the gray matter of the spinal cord. In chronic compressive myelopathy, the pathological changes in the gray matter of the spinal cord include loss of motor neurons, necrosis and cavitation (1,16,19). Therefore, EMG may potentially play specific roles during the course of CSM management (4).

In the present study, there was a significant association between ISI and EMG. In the 45 patients with an ISI on the T2-weighted MR images, 29 (64.44%) had corresponding enlarged MUAPs. As ISIs and enlarged MUAPs are able to reflect the pathological changes in the gray matter of the spinal cord, the two examinations were applied together to differentiate between the neural situations in the CSM patients. Based on the MRI/EMG classification system the patients were divided into four types: Type I, MRI/EMG (−/−); Type II, MRI/EMG (+/−); Type III, MRI/EMG (−/+); and Type IV, MRI/EMG (+/+). The patients underwent surgical intervention by anterior, posterior and posterior-anterior united decompression based on their cord compression ratio and other factors. Until the final follow-up, a different recovery ratio was observed between the four study groups. The surgical outcome was best in Type I and worst in Type IV patients, while the outcome for patients of Types II and III were observed in-between. The results revealed that the patients without ISIs or enlarged MUAPs were able to obtain a good prognosis.

Ramanauskas *et al* divided myelomalacia into three stages: early, intermediate and late (20). A change in the signal intensity of the spinal cord observed on MRI reflected cord edema in the early stage and cystic necrosis of the central gray matter following prolonged cord edema in the late stage. Zhang *et al* quantized the signal intensity ratio of the T2-weighted MRI and identified that an increase in signal intensity ratio was associated with older patients, longer disease duration and a trend for a reduced recovery rate (13). In the present study, relative index also had the tendency to have more severe symptoms and a worse prognosis. Type IV patients had a lower pre-operative JOA score, a longer disease duration and most significant of all, a worse post-operative recovery rate than patients of the other three types. These factors have previously been reported to affect the surgical outcome in patients with CSM (21–23).

In summary, these results indicate that there is a distinct correlation between EMG combined with MRI and the rate of clinical improvement in CSM patients. Patients who have a negative EMG and who lack an ISI tend to have mild symptoms, a short disease duration and experience a good prognosis. The grading system based on the EMG and MRI results is extremely thorough, appears to be objective for each observer and is beneficial for forming a prognosis of the surgical outcome of CSM.

## Figures and Tables

**Table I:** Disability classification of cervical spondylotic myelopathy (CMS).

Grade	Disability classification
0	Root signs and symptoms, no cord involvement
1	Signs of cord involvement, normal gait
2	Mild gait involvement, able to be employed
3	Gait abnormality prevents employment
4	Able to ambulate only with assistance
5	Chairbound or bedridden

**Table II:** Cervical spondylotic myelopathy (CSM) disability distribution in the four MRI/EMG patient types (number of patients).

	Grades of CSM disability						
MRI/EMG	0	1	2	3	4	5	Total
−/−	9	10	7	4	3	3	36
+/−	3	3	4	3	1	2	16
−/+	2	3	2	3	2	1	13
+/+	3	3	4	6	8	5	29
All	17	19	17	16	14	11	94

Kruskal-Wallis H test showing Hc=9.000, P=0.029. MRI, magentic resonance imaging; EMG, elecromyography.

**Table III:** Surgical outcomes in each EMG/MRI patient type (number of patients).

	Surgical outcome				
MRI/EMG	Excellent	Good	Fair	Poor	Total
−/−	26	7	2	1	36
+/−	7	4	3	2	16
−/+	5	3	3	2	13
+/+	3	8	12	6	29
All	41	22	20	11	94

Kruskal-Wallis H test showing Hc=27.463, P<0.001. MRI, magnetic resonance imaging; EMG, electromyography.

**Table IV:** Age, disease duration and pre-operative JOA score in each MRI/EMG patient type (mean ± SD).

	MRI/EMG					
Factor	−/−	+/−	−/+	+/+	Test statistic	P-value
Age (years)	53.17±5.79	52.32±13.61	50.60±7.95	54.43±10.06	F=2.352	0.131
Duration of disease (months)	7.15±3.20	10.24±2.20	9.57±3.64	16.43±6.20	F=7.830	0.004
Pre-operative JOA score	10.17±2.85	9.13±2.38	9.35±2.50	7.43±2.34	F=4.457	0.017

JOA, Japanese Orthopedic Association; MRI, magentic resonance imaging; EMG, electromyography.

**Table V:** Gender and cord compression ratio in each MRI/EMG patient type (number of patients).

	MRI/EMG					
Factor	−/−	+/−	−/+	+/+	Test statistic	P-value
Gender					χ^2^=0.540	0.910
Male	23	9	7	17		
Female	13	7	6	12		
Cord compression ratio					χ^2^=1.550	0.671
<1/3	15	9	6	11		
1/3-1/2	12	4	4	9		
>1/2	9	3	3	9		

MRI, magnetic resonance imaging; EMG, electromyography.
